# Photon-Counting CT Enhances Diagnostic Accuracy in Stable Coronary Artery Disease: A Comparative Study with Conventional CT

**DOI:** 10.3390/jcm14176049

**Published:** 2025-08-26

**Authors:** Mitsutaka Nakashima, Toru Miyoshi, Shohei Hara, Ryosuke Miyagi, Takahiro Nishihara, Takashi Miki, Kazuhiro Osawa, Shinsuke Yuasa

**Affiliations:** 1 Department of Cardiovascular Medicine, Okayama University Graduate School of Medicine, Dentistry and Pharmaceutical Sciences, Okayama 700-8558, Japan; mitsn1023@gmail.com (M.N.); hr5.sho24rh@gmail.com (S.H.); p0bz6moc@s.okayama-u.ac.jp (R.M.); taka.0204.hiro@gmail.com (T.N.); tm.f20c.2000@gmail.com (T.M.); yuasa@okayama-u.ac.jp (S.Y.); 2 Department of General Internal Medicine 3, Kawasaki Medical School General Medicine Centre, Okayama 700-8505, Japan; rohiwasa@yahoo.co.jp

**Keywords:** photon-counting CT, coronary CT angiography, diagnostic accuracy, invasive coronary angiography

## Abstract

**Background/Objectives:** Coronary CT angiography (CCTA) is a cornerstone in evaluating stable coronary artery disease (CAD), but conventional energy-integrating detector CT (EID-CT) has limitations, including calcium blooming and limited spatial resolution. Photon-counting detector CT (PCD-CT) may overcome these drawbacks through enhanced spatial resolution and improved tissue characterization. **Methods**: In this retrospective, propensity score–matched study, we compared CCTA findings from 820 patients (410 per group) who underwent either EID-CT or PCD-CT for suspected stable CAD. Primary outcomes included stenosis severity, high-risk plaque features, and downstream invasive coronary angiography (ICA) referral and yield. **Results**: The matched cohorts were balanced in demographics and cardiovascular risk factors (mean age 67 years, 63% male). PCD-CT showed a favorable shift in stenosis severity distribution (*p* = 0.03). High-risk plaques were detected less frequently with PCD-CT (22.7% vs. 30.5%, *p* = 0.01). Median coronary calcium scores did not differ (*p* = 0.60). Among patients referred for ICA, those initially evaluated with PCD-CT were more likely to undergo revascularization (62.5% vs. 44.1%), and fewer underwent potentially unnecessary ICA without revascularization (3.7% vs. 8.0%, *p* = 0.001). The specificity in diagnosing significant stenosis requiring revascularization was 0.74 with EID-CT and 0.81 with PCD-CT (*p* = 0.04). **Conclusions**: PCD-CT improved diagnostic specificity for CAD, reducing unnecessary ICA referrals while maintaining detection of clinically significant disease. This advanced CT technology holds promise for more accurate, efficient, and patient-centered CAD evaluation.

## 1. Introduction

Coronary artery disease (CAD) is a leading cause of morbidity and mortality globally, and noninvasive imaging plays a central role in the diagnostic evaluation of patients with stable chest pain [1]. Coronary CT angiography (CCTA) is widely recommended due to its high sensitivity and negative predictive value for detecting coronary atherosclerosis [2]. However, traditional CCTA performed using energy-integrating detector CT (EID-CT) is limited by moderate spatial resolution and blooming artifacts from calcified plaques, which may lead to overestimation of stenosis severity [3]. These limitations can contribute to misclassification under the Coronary Artery Disease Reporting and Data System (CAD-RADS) [4], potentially triggering unnecessary invasive coronary angiography (ICA).

Photon-counting detector CT (PCD-CT) is a next-generation imaging technology that directly detects X-ray photons, offering significantly improved spatial resolution, higher contrast-to-noise ratio, and intrinsic spectral imaging capabilities [5]. These advantages are expected to reduce the impact of calcification artifacts, better visualize the coronary lumen, and provide more precise plaque characterization. Initial studies have shown that PCD-CT can result in reclassification of stenosis severity and a reduction in false-positive assessments of obstructive CAD [6,7,8,9,10,11,12]. In parallel, the enhanced image quality may influence the detection of high-risk plaque features, though its net clinical effect remains under evaluation.

In our institution, the PCD-CT system (Naeotom Alpha; Siemens Healthcare GmbH, Erlangen, Germany) was introduced in December 2022, enabling real-world clinical evaluation of this technology. Our previous study suggested that PCD-CT could provide additional diagnostic value in challenging settings, including patients with extensive calcifications or prior stent implantation, where conventional CT often struggles [11].

This study aimed to compare the diagnostic utility of PCD-CT and EID-CT in patients with suspected stable CAD. We hypothesized that PCD-CT would result in fewer patients being classified with obstructive CAD or high-risk plaque and would reduce downstream ICA referrals without compromising diagnostic sensitivity. Using a propensity-matched cohort, we evaluated CAD-RADS-based stenosis severity, the prevalence of high-risk plaques, and the rates and outcomes of ICA referrals to assess the impact of CT modality on diagnostic accuracy and patient management.

## 2. Materials and Methods

### 2.1. Study Design and Population

This was a retrospective, single-center comparative study of two CT technologies for coronary angiography. We included consecutive patients with suspected stable CAD (typically presenting with stable chest pain or positive stress tests) who underwent CCTA between January 2021 and December 2024. Two different CT scanner types were used during this period at our institution: a conventional 2 × 128-slice dual-source CT scanner with EID-CT, and a latest-generation dual-source CT scanner equipped with PCD-CT. We applied the following exclusion criteria to the cohort: a history of coronary revascularization (prior percutaneous coronary intervention [PCI] or coronary artery bypass grafting [CABG]), known coronary anomalies, vasculitis, congenital coronary artery disease, and any CCTA studies with nondiagnostic image quality. These exclusions ensured that we evaluated only new diagnostic CCTA exams in patients without prior surgical or stent alterations of coronary anatomy.

### 2.2. Patient Selection and Matching

**Figure 1:**
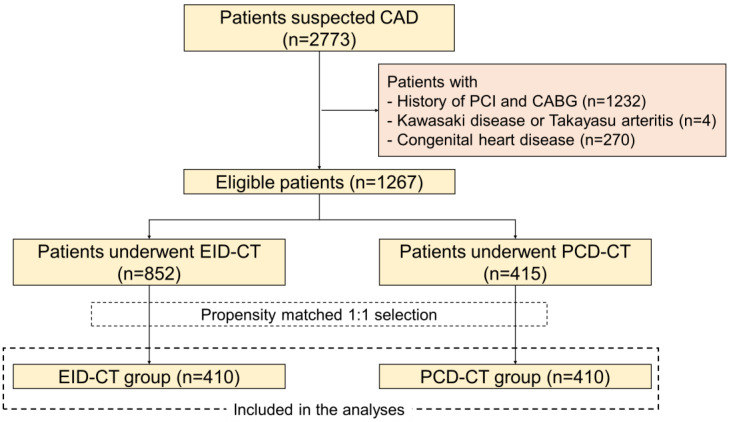
Flow diagram of this study. Among 2773 patients with suspected coronary artery disease (CAD) who underwent coronary CT angiography (CCTA), 1267 patients met the inclusion criteria and were evaluated. Patients who underwent either energy-integrating detector CT (EID-CT) or photon-counting detector CT (PCD-CT) were selected and matched using propensity score matching. Ultimately, 410 patients were included in each of the EID-CT and PCD-CT groups for the final analyses. CABG, coronary artery bypass grafting; CAD, coronary artery disease; CT, computed tomography; EID-CT, energy-integrating detector computed tomography; PCD-CT, photon-counting detector computed tomography; PCI, percutaneous coronary intervention.

**Figure 2:**
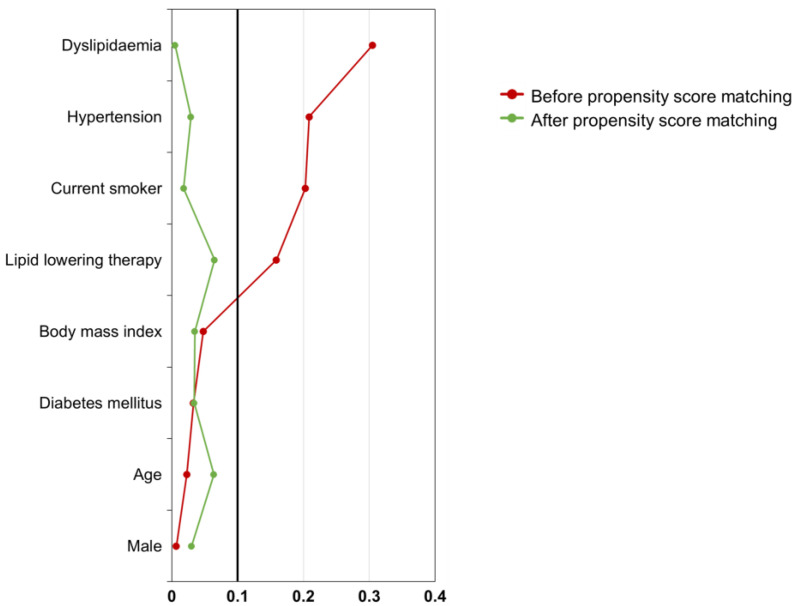
SMDs of Variables Before and After Propensity Score Matching. The red line represents the SMDs of baseline variables before matching, and the green line represents those after matching. Following propensity score matching, the SMDs for all variables decreased and fell below the threshold of 0.1, indicating adequate covariate balance between groups. SMD, standardized mean difference.

A total of 2773 patients underwent CCTA for suspected CAD during the study period. Of these, 1506 patients were excluded due to the criteria above (including 1232 with prior PCI/CABG, 4 with inflammatory arterial disease, and 270 with congenital heart disease), leaving 1267 patients with eligible CCTA studies (Figure 1). Among the eligible cohort, 852 patients had been scanned on the EID-CT system and 415 patients on the PCD-CT system. Given the differences in baseline characteristics between these groups (reflecting, for example, that early adopters of PCD-CT were a subset and had lower rates of certain risk factors), we implemented propensity score matching to allow a fair comparison of diagnostic outcomes. The propensity model included age, sex, and major cardiovascular risk factors (hypertension, diabetes mellitus, dyslipidemia, smoking status, and use of lipid-lowering therapy) as covariates. Propensity-score matching without replacement was performed in a 1:1 ratio (PCD-CT:EID-CT) with a caliper of 0.2 standard deviations of the propensity score. This yielded two matched groups of 410 patients each. The balance of baseline covariates after matching was assessed by standardized mean differences (SMD), with an SMD < 0.1 taken to indicate a negligible difference between groups (Figure 2) [13].

### 2.3. CT Angiography Acquisition and Analysis

**Figure 3:**
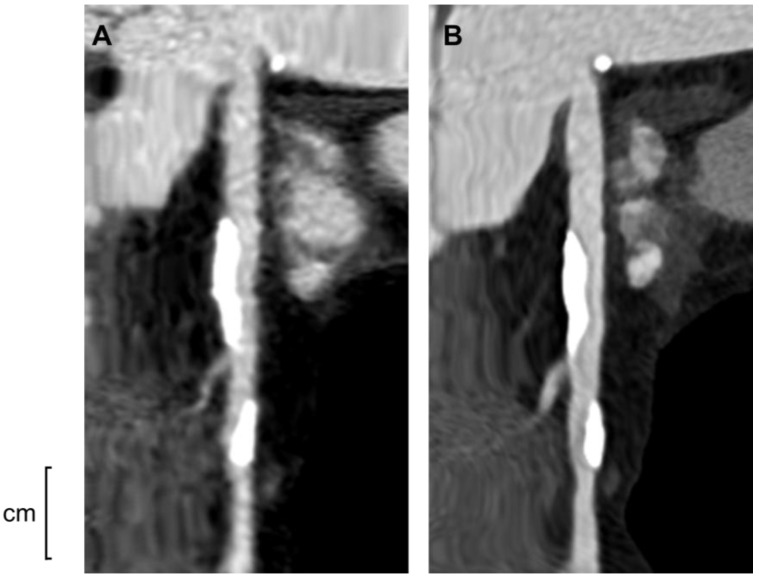
Representative coronary CT angiography images of the right coronary artery (RCA) from the same patient, obtained using (**A**) energy-integrating detector CT (EID-CT) and (**B**) photon-counting detector CT (PCD-CT). PCD-CT enabled clearer visualization of coronary artery calcifications compared to EID-CT, owing to its superior spatial resolution and reduced blooming artifacts.

All patients underwent CCTA with standardized protocols for coronary imaging. EID-CT scans were performed with a conventional dual-source CT (Somatom Definition Flash; Siemens Healthcare GmbH, Erlangen, Germany) using standard spatial resolution mode (detector collimation 2 × [128 × 0.6 mm]) and iterative image reconstruction. PCD-CT scans were performed on a photon-counting scanner (Naeotom Alpha; Siemens Healthcare GmbH, Erlangen, Germany) with similar acquisition parameters (detector collimation, 2 × [144 × 0.4 mm]), with the option of ultra-high-resolution mode (collimation up to 2 × [120 × 0.2 mm]) applied for coronary imaging. Radiation dose reduction techniques (prospective ECG gating, tube current modulation) and contrast injection protocols were employed per routine clinical standards for coronary CTA (Figure 3). All CCTA images were analyzed on a dedicated workstation by experienced cardiologists blinded to patient outcomes. Coronary stenoses were evaluated in all major segments. For each patient, the highest grade of coronary stenosis identified (per cent diameter stenosis) was recorded and categorized into one of five ordinal severity categories: <25% (no significant stenosis or minimal plaque), 25–49% (mild stenosis), 50–69% (moderate stenosis), 70–99% (severe stenosis), or 100% (total occlusion). These categories correspond to the CAD-RADS 2.0 reporting scheme (CAD-RADS scores 0–1, 2, 3, 4, and 5, respectively) [4] and were used to compare the overall stenosis burden between groups. We also assessed the presence of “high-risk plaque” features on CCTA. In accordance with Society of Cardiovascular CT guidelines, a plaque was considered high-risk if it demonstrated adverse features suggestive of vulnerability, such as positive remodeling, low attenuation plaque core (<30 HU), napkin-ring sign, or spotty calcifications [4]. Patients were labeled as having high-risk plaque if any coronary plaque with one or more of these features was present on the scan. Additionally, the coronary artery calcium score (CACS) was measured from the non-contrast CT acquisition, in which coronary artery calcium in epicardial coronary arteries was assessed in 3.0 mm slices throughout the coronary artery regions, and the Agatston method was calculated [14]. The CACS was categorized using the cut-off of 0,100,300, and 1000 [15].

### 2.4. Clinical Endpoints

We extracted data on downstream diagnostic and therapeutic interventions from the patients’ medical records, with particular attention to ICA referrals and coronary revascularization. Decisions for ICA were made by treating physicians based on the CCTA findings combined with clinical assessment, following standard guidelines (generally, a CCTA showing ≥70% stenosis in a major vessel or other high-risk findings prompted direct ICA, whereas 50–69% stenosis often led to either functional testing or ICA on a case-by-case basis). We recorded whether each patient underwent an ICA within 3 months of the CCTA. Furthermore, among patients who underwent ICA, we determined if a revascularization procedure (PCI or CABG) was ultimately performed, as a proxy for a true positive finding that altered patient management. These outcome measures allowed us to evaluate the clinical impact of PCD-CT vs. EID-CT in terms of how often the imaging findings translated into necessary invasive interventions. Additionally, we investigated the clinical composite outcome: cardiac death, myocardial infarction, urgent revascularization, and hospitalization for heart failure after the CT scan.

### 2.5. Statistical Analysis

Continuous variables are presented as mean ± standard deviation if normally distributed, or median with interquartile range (IQR) if skewed. Categorical variables are presented as counts and percentages. Baseline characteristics and imaging findings were compared between the EID-CT and PCD-CT groups using appropriate statistical tests. The chi-square test (or Fisher’s exact test for low cell counts) was used for categorical comparisons. Continuous variables were compared with Student’s *t*-test for independent samples if normally distributed, or the Mann–Whitney U test if non-normal. The distribution of stenosis severity categories between groups was compared using the chi-square test for trend. The effectiveness of propensity matching was confirmed by checking that no baseline covariate had a significant residual difference. To assess the independent association of scanner type (EID vs. PCD) with the likelihood of downstream ICA, a logistic regression analysis was also performed on the matched cohort, adjusting for potential confounders including age, sex, hypertension, hyperlipidemia, and calcium score. The clinical composite outcome was compared between the groups using the log-rank test. All statistical analyses were conducted using SPSS version 25 (IBM Corp., Armonk, NY, USA) and R version 4.3.2 (R Foundation for Statistical Computing, Vienna, Austria). A two-sided *p*-value of <0.05 was considered statistically significant.

## 3. Results

### 3.1. Patient Population

After applying exclusion criteria, 1267 patients who underwent CCTA for suspected stable CAD were identified. Of these, 852 patients underwent CCTA using EID-CT, while 415 patients underwent PCD-CT. Following 1:1 propensity score matching, 410 well-balanced pairs were identified. The matched cohorts were comparable in age (mean approximately 68 years), sex (63% male), and cardiovascular risk factors, including hypertension, diabetes mellitus, dyslipidemia, and smoking status (all *p* > 0.3; SMD < 0.1) (Table 1).

### 3.2. Coronary CTA Findings

**Figure 4:**
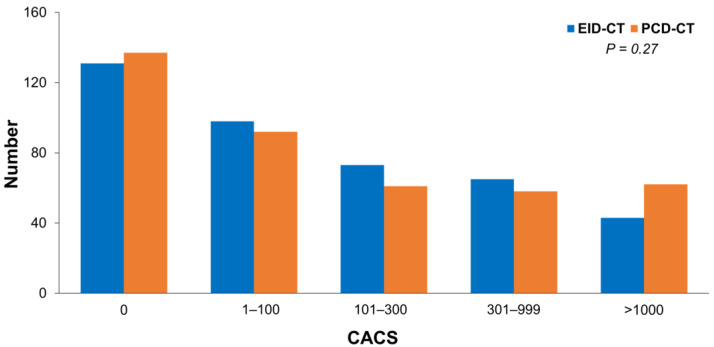
Comparison of EID-CT and PCD-CT distributions across CACS categories. EID-CT, energy-integrating detector computed tomography; PCD-CT, photon-counting detector computed tomography; CACS, coronary artery calcium score.

The median computed tomography dose index volume (CTDIvol) was 72.5 mGy in the EID-CT group and 46.4 mGy in the PCD-CT group, and the median dose length product (DLP) was 1801 mGy·cm and 1325 mGy·cm, respectively. The prevalence of obstructive CAD was significantly lower in the PCD-CT group compared with the EID-CT group (Table 2). The frequency of severe (70–99%) and intermediate-grade stenoses (50–69%) was lower in the PCD-CT group (15.6% vs. 12.9% and 9.3% vs. 12.9%, respectively). The overall distribution of stenosis severity differed significantly between the two groups (*p* = 0.033). Moreover, high-risk plaque features were less frequently identified with PCD-CT than with EID-CT (22.7% vs. 30.5%; *p* = 0.014). Coronary artery calcium scores were similar between the groups (*p* = 0.60). The distributions of EID-CT and PCD-CT were not significantly different across CACS categories (Figure 4).

### 3.3. Downstream ICA and Outcomes

**Figure 5:**
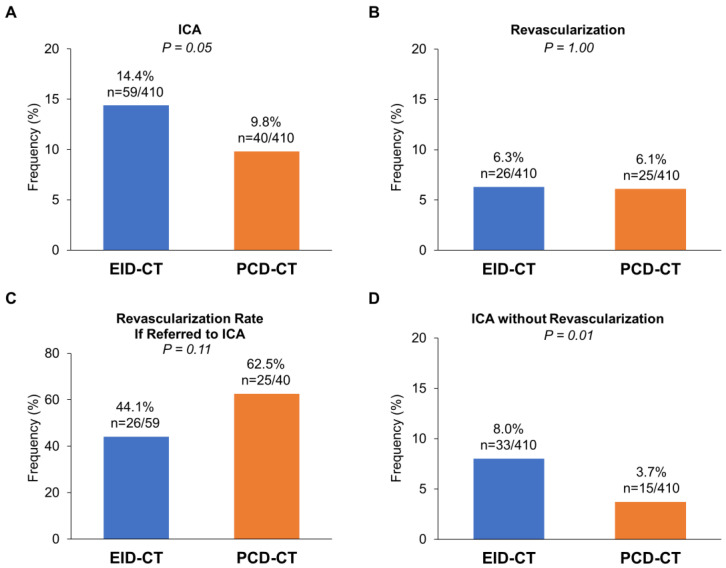
Clinical Impact of PCD-CT on Downstream Management. (**A**) Rates of referral to invasive coronary angiography (ICA) following coronary CT angiography. (**B**) Overall revascularization rates. (**C**) Proportion of patients undergoing revascularization among those referred to ICA. (**D**) Proportion of patients undergoing ICA without subsequent revascularization. EID-CT, energy-integrating detector computed tomography; PCD-CT, photon-counting detector computed tomography; ICA, invasive coronary angiography.

Referral to ICA was less frequent following PCD-CT compared to EID-CT (9.8% vs. 14.4%; *p* = 0.05) (Figure 5A). Multivariable logistic regression analysis demonstrated that, after adjusting for potential confounders, undergoing PCD-CT was significantly associated with lower odds of referral to ICA compared with EID-CT (odds ratio: 0.465; 95% confidence interval: 0.283–0.765). Of all, the rate of revascularization was similar between the PCD-CT group and the EID-CT group (*p* = 1.00) (Figure 5B). Among patients referred to ICA, the revascularization rate tended to be higher in the PCD-CT group (62.5% vs. 44.1%), although this difference did not reach statistical significance (*p* = 0.11) (Figure 5C). Notably, the proportion of patients undergoing ICA without subsequent revascularization—considered potentially unnecessary diagnostic ICA—was significantly lower in the PCD-CT group compared to the EID-CT group (3.7% vs. 8.0%; *p* = 0.001) (Figure 5D). In diagnosing significant stenosis requiring revascularization, the sensitivity was 1.0 in both groups, and the specificity in diagnosing significant stenosis requiring revascularization was 0.74 in the EID-CT group and 0.81 in the PCD-CT group (*p* = 0.04), and the positive predictive value was 0.21 in the EID-CT group and 0.25 in the PCD-CT group (*p* = 0.56). Multivariable logistic regression analysis further showed that undergoing PCD-CT was significantly associated with lower odds of referral to ICA without subsequent revascularization (odds ratio: 0.361; 95% confidence interval: 0.184–0.707), after adjusting for potential confounders. During a median follow-up period of 2.2 years, there was no difference between the two groups in the clinical composite outcome after the CT scan (19 patients in the EID-CT group and 11 patients in the PCD-CT group, *p* = 0.09).

## 4. Discussion

In this propensity-matched comparative study of PCD-CT versus EID-CT for the evaluation of CAD, we found that PCD-CT offers significant diagnostic advantages. The key finding was that PCD-CT scans resulted in fewer patients being labeled with obstructive CAD or high-risk plaque, and consequently fewer ICA were recommended, without evidence of missed significant lesions. These results support the concept that PCD-CT improves the specificity of CCTA in stable CAD patients and can enhance clinical decision-making by improving the specificity in diagnosing significant stenosis requiring revascularization.

Animal studies have demonstrated that PCD-CT reduces blooming artifacts from calcified coronary lesions, leading to more accurate stenosis assessment and providing superior delineation of coronary plaque morphology, improving the distinction between calcified and non-calcified components relative to EID-CT [16,17]. Previous reports have shown that the spatial resolution advantage of PCD-CT is most pronounced in the in-plane direction, whereas improvements in the *z*-axis resolution are more dependent on collimation and reconstruction settings [5,18,19]. This anisotropy is clinically relevant, as coronary imaging strongly relies on high in-plane resolution to delineate small luminal changes, in contrast to organs such as the lung or liver, where volumetric depiction plays a larger role [8,11]. Clinical research has also shown that PCD-CT can provide a better diagnostic performance for obstructive CAD [6,7,8,9,10,11,12]. Our study contributes to the expanding literature demonstrating that the technical advances of PCD-CT result in clinically meaningful benefits. Specifically, the higher spatial resolution of PCD-CT allows more accurate stenosis assessment. We observed a shift toward lower stenosis severity grades in the PCD-CT group: for example, lesions that might have been moderate (50–69%) on EID-CT were often measured as mild (<50%) on PCD-CT. This aligns with prior investigations in which side-by-side imaging on EID vs. PCD systems showed PCD-CT consistently downgrading stenosis severity. Koons et al. reported an average 11% reduction in diameter stenosis measurement using PCD-CT, causing over one-third of lesions to drop into a less severe category [7]. In our data, the net effect was a roughly 20% relative reduction in the number of patients meeting criteria for obstructive CAD on PCD-CT vs. EID-CT (24% vs. 30%). This is a clinically significant difference, as it implies that many patients with borderline findings by older generation CT might be reclassified as having non-obstructive disease when imaged with PCD technology. From a patient management perspective, those reclassified patients often avoid unnecessary invasive angiography or other tests. Our matched analysis reinforces that it was the imaging modality—rather than patient risk factors—driving this difference in referral rates. In the Japanese healthcare system, the medical costs for a CT scan are the same regardless of the EID-CT or the PCD-CT. Therefore, the additional medical economic burden from switching to PCD-CT has not increased. Additionally, since the number of ICA without revascularization has decreased, it can be said that there is a cost–benefit from using PCD-CT. Regarding radiation exposure, PCD-CT does not require a prolonged operation time and a higher dose per scan compared to EID-CT, and since it reduces the number of ICA without revascularization, the risk is even lower. Furthermore, no difference was observed in long-term prognosis after the CT scan. This suggests that the reduction in ICA without revascularization did not lead to an increase in poor outcomes.

Importantly, the reduction in downstream invasive procedures associated with PCD-CT did not result in a loss of diagnostic sensitivity. In our cohort, PCD-CT detected coronary artery disease, including ≥50% stenoses, with accuracy comparable to that of EID-CT. This observation is in line with previous studies. For example, a large registry analysis by Sakai et al. demonstrated that PCD-CT preserved a high sensitivity of around 91% while significantly reducing false-positive findings, which led to a higher positive predictive value for obstructive coronary disease [10]. In our study, we also observed a trend toward a higher revascularization rate among patients undergoing ICA after PCD-CT (62.5%) compared with EID-CT (44.1%). Although this difference did not reach statistical significance, it reflects the 8% absolute increase in revascularization observed with PCD-CT in the aforementioned registry (43% vs. 35%). Taken together, these results suggest that PCD-CT may improve the efficiency of the diagnostic process by reducing unnecessary invasive testing while increasing the likelihood that patients referred for ICA truly require treatment.

The observed difference in high-risk plaque detection between EID-CT and PCD-CT is also noteworthy. We found about one-third fewer patients with “high-risk” plaque features on PCD-CT scans compared to EID-CT scans. This could be interpreted in several ways. One possibility is that PCD-CT’s better image quality allows more confident discrimination of plaque features, filtering out some false-positive interpretations of high-risk features that might occur on grainier EID-CT images [17]. For example, what might appear as a low-attenuation core within a plaque on a standard CT might actually be partial volume averaging or noise, which is clarified on PCD-CT as a more moderate attenuation plaque without a true necrotic core. Similarly, positive remodeling or napkin-ring sign might be overcalled on lower-resolution images. Thus, PCD-CT may improve the specificity of high-risk plaque identification, analogous to its effect on stenosis grading. High-risk plaque features on CCTA have been associated with future acute coronary events [20], but their presence can also trigger more aggressive management upfront. If PCD-CT more selectively identifies truly high-risk plaques, this could help focus preventive therapies and further testing on the right subset of patients. Future studies with longer-term follow-up could examine whether PCD-CT’s characterization of plaque risk correlates better with outcomes than EID-CT’s does. On the other hand, no difference in CACS was found between the EID-CT group and the PCD-CT group. This result may suggest that while PCD-CT is expected to reduce artifacts caused by calcium, it does not underestimate the amount of calcification or substantially affect the reference values of the Agatston score that have been established in previous studies.

It is also worth mentioning that PCD-CT brings other potential benefits not directly measured in our study. PCD-CT can improve image quality in obese patients and may allow reduced contrast doses due to better contrast-to-noise performance [5]. Moreover, ongoing developments in PCD technology could further enhance temporal resolution and spectral imaging capabilities, opening new frontiers such as plaque composition analysis (e.g., distinguishing lipid-rich from fibrotic plaques) and even myocardial perfusion imaging within the same scan. While our study focused on the core diagnostic performance for lumen stenosis and plaque features, these additional advantages could further contribute to improved diagnostic utility in the future.

We acknowledge several limitations of this study. First, the design was observational and retrospective, with the inherent possibility of selection bias. Although we used propensity matching to equate the groups on measured confounders, there may still be unmeasured differences (for instance, subtle differences in referral patterns or scanner protocols over time) that influenced the outcomes. The PCD-CT scans were performed slightly later in the study period, which might coincide with evolving operator experience or other temporal trends independent of the technology. Second, this study was conducted in a single center in East Asia, and the majority of the participants were East Asian. There is a potential selection bias, especially regarding racial differences in the prevalence and risk profile of CAD. Additionally, because our institution is a general hospital, there is a possibility that the patient population may include more cases with a history of non-cardiac diseases, such as malignant tumors, than the general population. Third, our analysis of invasive angiography yield was limited to those who underwent ICA, and we did not systematically verify all CT findings against an ICA gold standard in patients who were not referred. Thus, we primarily assessed differences in clinical management and presumed diagnostic accuracy rather than performing a head-to-head per-patient sensitivity/specificity calculation. Fourth, we did not directly measure image quality metrics or radiation dose in this study. Other works have shown modest dose reduction with PCD-CT due to its higher detector efficiency [12], but we cannot draw conclusions on that from our data. Lastly, our findings reflect a single-center experience with specific CT scanner models (first-generation PCD-CT and a high-end EID-CT); results might differ with other scanner platforms or in other clinical settings.

## 5. Conclusions

Our comparative analysis demonstrates that PCD-CT outperforms conventional CT in key diagnostic aspects for stable coronary artery disease. PCD-CT markedly reduces the overestimation of stenosis and the identification of ambiguous high-risk plaque, which, in turn, lowers the rate of unwarranted invasive angiography. As a result, PCD-CT has the potential to improve patient care by ensuring that invasive evaluations and interventions are better targeted to those who truly need them. This technology thus holds promise for refining clinical pathways in cardiovascular care by enhancing diagnostic precision and ultimately improving patient outcomes. Future studies should build on these findings to guide the broader implementation of PCD-CT into routine practice, evaluate its long-term effects on patient outcomes, and more precisely quantify its cost–benefit profile in coronary artery disease management.

## Figures and Tables

**Table 1:** Baseline clinical characteristics of this study.

	Before Propensity Score Matching			After Propensity Score Matching		
Variables	EID-CT	PCD-CT	SMD	EID-CT	PCD-CT	SMD
N	852	415		410	410	
Age, years	66.9 ± 12.9	67.2 ± 13.4	0.02	68.0 ± 12.5	67.1 ± 13.3	0.06
Male	531 (62.3)	260 (62.7)	<0.01	263 (64.1)	257 (62.7)	0.03
Body mass index, kg/m^2^	24.2 ± 4.5	24.5 ± 6.9	0.05	24.3 ± 4.5	24.2 ± 4.4	0.04
Hypertension	535 (62.8)	218 (52.5)	0.21	224 (54.6)	218 (53.2)	0.03
Diabetes mellitus	234 (27.5)	108 (26.0)	0.03	99 (24.1)	105 (25.6)	0.03
Dyslipidaemia	394 (46.2)	131 (31.6)	0.31	132 (32.2)	131 (32.0)	<0.01
Current smoker	127 (14.9)	35 (8.4)	0.20	33 (8.0)	35 (8.5)	0.02
Lipid-lowering therapy	316 (37.1)	123 (29.6)	0.16	109 (26.6)	121 (29.5)	0.07

Data are presented as numbers (%), mean ± standard deviation. EID-CT, energy-integrating detector–computed tomography; PCD-CT, photon-counting detector–computed tomography; SMD, standardized mean difference.

**Table 2:** Baseline clinical characteristics of this study.

c	EID-CT (N = 410)	PCD-CT (N = 410)	*p* Value
Highest stenosis severity			0.033
<25% (CAD-RAD 0–1)	222 (54.1)	244 (59.5)	
25–49% (CAD-RAD 2)	63 (15.4)	66 (16.1)	
50–69% (CAD-RAD 3)	53 (12.9)	38 (9.3)	
70–99% (CAD-RAD 4)	64 (15.6)	53 (12.9)	
100% (CAD-RAD 5)	8 (2.0)	9 (2.2)	
High-risk plaque	125 (30.5)	93 (22.7)	0.014
CACS	55 (0, 334)	65 (0, 435)	0.60

Data are presented as the number (%) or median (25th, 75th percentile). CACS, coronary artery calcium score.

## Data Availability

The data presented in this study are available upon request from the corresponding author. The data is not publicly available due to privacy concerns.
